# Social Dysfunction and Diet Outcomes in People with Psychosis

**DOI:** 10.3390/nu9010080

**Published:** 2017-01-19

**Authors:** Doreen Mucheru, Mary-Claire Hanlon, Linda E. Campbell, Mark McEvoy, Lesley MacDonald-Wicks

**Affiliations:** 1 Faculty Health and Medicine, The University of Newcastle, Callaghan 2308, Australia; Doreen.Mucheru@uon.edu.au (D.M.); Mary-Claire.Hanlon@uon.edu.au (M.-C.H.); mark.mcevoy@newcastle.edu.au (M.M.); 2 Brain and Mental Health Program, Hunter Medical Research Institute, New Lambton 2305, Australia; 3 Priority Research Centre for Brain and Mental Health, Mater Hospital, Waratah 2298, Australia; 4 Department of Radiation Oncology, Calvary Mater Newcastle, Waratah 2298, Australia; 5 Hunter Cancer Research Alliance, The University of Newcastle, Callaghan 2308, Australia; 6 Faculty of Science, The University of Newcastle, Callaghan 2308, Australia; linda.e.campbell@newcastle.edu.au; 7 Priority Research Centre GrowUpWell, The University of Newcastle, Callaghan 2308, Australia; 8 Centre for Clinical Epidemiology & Biostatistics, Hunter Medical Research Institute, New Lambton 2305, Australia; 9 Priority Research Centre for Physical Activity and Nutrition, The University of Newcastle, Callaghan 2308, Australia

**Keywords:** psychosis, social isolation, social dysfunction, fruit intake, vegetable intake, food security, diet

## Abstract

This analysis aimed to examine the association of social dysfunction with food security status, fruit intake, vegetable intake, meal frequency and breakfast consumption in people with psychosis from the Hunter New England (HNE) catchment site of the Survey of High Impact Psychosis (SHIP). Social dysfunction and dietary information were collected using standardised tools. Independent binary logistic regressions were used to examine the association between social dysfunction and food security status, fruit intake, vegetable intake, meal frequency and breakfast consumption. Although social dysfunction did not have a statistically significant association with most diet variables, participants with obvious to severe social dysfunction were 0.872 (95% CI (0.778, 0.976)) less likely to eat breakfast than those with no social dysfunction *p* < 0.05. Participants with social dysfunction were therefore, 13% less likely to have breakfast. This paper highlights high rates of social dysfunction, significant food insecurity, and intakes of fruits and vegetables below recommendations in people with psychosis. In light of this, a greater focus needs to be given to dietary behaviours and social dysfunction in lifestyle interventions delivered to people with psychosis. Well-designed observational research is also needed to further examine the relationship between social dysfunction and dietary behaviour in people with psychosis.

## 1. Introduction

Social isolation and loneliness are some of the biggest self-reported challenges faced by people living with psychosis; in the Survey of High Impact Psychosis (SHIP), more than one third of people with psychosis stated that social isolation was one of their biggest concerns for the coming year with two-thirds having never been married and also reporting that their illness made it difficult to maintain close family and social ties [1,2]. Linz and Sturm [3] defined social isolation as unintentional aloneness without a fulfilling social connection, which gives rise to subjective loneliness and distress [3]. Social isolation is integral to the experience of social dysfunction [4]. Social dysfunction is an umbrella term for the restriction of participation in social relationships based on an evaluation of interpersonal relationships [4]. Social isolation is therefore a marker of social dysfunction [4]. The International Statistical Classification of Diseases and Related Health Problems (ICD) describes psychosis as the experience of hallucinations, delusions or gross excitement and overactivity, psychomotor retardation and catatonic behaviour that causes distress and interferes with personal functions [5]. The experience of psychotic symptoms is common in diagnoses which include schizophrenia type disorders ((ICD) codes F20-F29) and mood or affective disorders ((ICD) codes F30-F39) [5]. Hawthorne [6] found that the prevalence of social isolation in the general Australian community was only 9%; highlighting that people living with psychosis in Australia are three times more likely to suffer social isolation and any of its negative correlates. It is well recognised that social participation is protective against disease, enhances coping with stress and improves disease outcomes, whereas social isolation results in the converse [7].

The overarching themes of social isolation in people with psychosis include: stigma, alienation and loneliness [3]. Stigma in people with psychosis occurs because they are viewed differently by society and, sometimes as a consequence, view themselves differently, thus hindering social integration and immersing themselves deeper into social isolation [8,9]. Alienation, on the other hand, occurs when individuals feel estranged from their social environment, which can result in further social withdrawal [10,11]. Loneliness is the subjective feeling of aloneness even when in a social situation [12]. The compounded effect of stigma, alienation and loneliness may thus predispose persons with psychosis to a greater degree of social isolation which directly contributes to depression and indirectly contributes to heart disease and increased overall mortality; all of which are issues of major concern for people with psychosis [2,13,14,15,16].

Social ties have been shown to influence health behaviours both positively and negatively [17]. Studies from the UK and USA show that social isolation negatively affects the quality and quantity of diet in the older population [18,19]. Further, a Canadian study by Tarasuk [20] showed women seeking food assistance, who reported feeling socially isolated, had higher odds of reporting food insecurity with severe or moderate hunger than those who did not report social isolation. Food insecurity occurs when there is limited access to nutritious foods or when there is a limitation in the ability to acquire these foods in ways that are socially acceptable [21]. Hence, food insecurity may reflect financial difficulty or challenges in acquiring safe and nutritionally adequate foods [21]. It is unclear what role social isolation has in the experience of food insecurity, however it is possible that people who function better socially are more likely to garner employment therefore giving them a financial advantage [22].

The interest in social isolation, and the potential effects it may have on one’s lifestyle, is growing [23]. Social isolation is now a strong predictor of mortality parallel to conventional risk factors such as hypertension, diabetes, smoking and excessive alcohol consumption [23,24]. Social isolation impacts mortality directly through the negative impact it has on chronic inflammation and indirectly through the effect it has on modifiable health behaviours [17,25,26]. A poor diet low in fruits and vegetables is an example of a modifiable health behaviour that is associated with social isolation [18,19]. People with psychosis in Australia report poor nutrition with as many as 71% consuming only one or less serves of fruit and 48% consuming only one or less serves of vegetables a day [2]. In recent years, research has found that health behaviours tend to co-occur; low fruit and vegetable intake has been linked to irregular meal and breakfast consumption in adolescents [27,28,29]. It is therefore important to assess whether social isolation is also associated with other dietary patterns that are linked to fruit and vegetable intake [29].

The current study measured social isolation as part of a social dysfunction assessment therefore the latter measure will be the focus of this study. Presently, there are no studies investigating the relationship between objectively measured social dysfunction, food security status, fruit intake, vegetable intake, breakfast consumption and meal frequency in people with psychosis, despite a high prevalence of social dysfunction in this population and their poor quality diet [2,30]. The aim of this study is therefore to examine the association of social dysfunction with food security status, fruit intake, vegetable intake, breakfast consumption and meal frequency of people with psychosis from the Hunter New England (HNE) catchment site of the SHIP. Additional diet outcomes from the SHIP such as frequency of adding salt to food and type of milk consumed were excluded from this analysis. This is because currently available research does not lead us to believe that a relationship between social isolation and these outcomes would be present [18,19,20,27].

## 2. Materials and Methods

This is a cross-sectional analysis of data from a cohort of Australians living with psychosis from the HNE who took part in the SHIP.

### 2.1. The Survey of High Impact Psychosis (SHIP)

The SHIP covered seven catchment areas. Inclusion criteria for the SHIP study were: adults between 18 and 64 years with psychotic symptoms, residing in any of the catchment sites and being in contact with public specialised mental health services (MHSs) or Community Management Organisations (CMOs, formerly known as Non-government organisations) funded to support people with mental illness. Exclusion criteria were: residing in a nursing home or prison, or inability to speak English sufficiently to answer all questions (whether due to English being a second language, or to intelligence quotient (IQ) of 70 or under).

The sampling technique used in the SHIP consisted of two phases; phase 1 involved a census of those in contact with public MHSs and CMOs supporting people with mental illness in March 2010, as well as a medical record review and census of those in contact with public MHSs 11 months prior to March (*n* = 7955). Individuals experiencing symptoms of psychosis identified from phase 1 were randomly selected to participate in phase 2 (*n* = 1825), which involved a full diagnostic interview, fasting blood tests, physical checks and cognitive functioning assessments with participants stratified by site and age group. By design, all participants met screening criteria for psychosis, however not everyone met the full diagnostic criteria for an ICD-10 non-organic psychotic disorder [2].

The SHIP was administered by trained mental health professionals, and was multi-faceted, comprising of interviews, fasting blood tests, physical checks and cognitive functioning assessments. The detailed methodology of the SHIP has been published online and in various peer-reviewed publications [2]. The HNE catchment site data were the focus of this analysis. HNE has a total area of about 62,000 km^2^ and a total population of about 1.5 million aged between 18 and 64 years; representative of 10% of total Australians in the same age bracket. The HNE catchment site includes mental health services (MHSs) in Lake Macquarie, Greater Newcastle and the Lower Hunter Valley, Australia.

### 2.2. Participants

Data from all 221 participants living with psychosis from the HNE cohort of the SHIP were analysed.

### 2.3. Measures

#### 2.3.1. Demographic Variables (SHIP Questions 0.04, 1.07, 11.02, 11.08, 0.05, 1.09, 2.04)

Demographic factors included: sex (male/female), Aboriginal or Torres Strait Islander descent (yes/no), marital status (single or never married/married or de facto/separated or divorced/widowed) government pension as the main source of income (yes/no), highest education qualification (left school with no qualifications/secondary school qualification or leaving certificate/tertiary certificate/bachelor’s degree/postgraduate qualifications/other) and income per fortnight (AU$300 or less/AU$300–AU$499/AU$500–AU$799/AU$800–AU$1000/AU$1000 or more). Age was calculated from the date of birth given.

#### 2.3.2. Diagnosis (SHIP Section 20)

Diagnoses were made by trained mental health professionals who held at minimum a bachelor’s degree within various fields in health, using the Diagnostic Module of the Diagnostic Interview for Psychosis (DIP-DM). The DIP-DM is a semi-structured clinical interview with good inter-rater reliability and excellent diagnostic validity [31]. An ICD-10 diagnosis was obtained by entering scores from the DIP-DM into a computer algorithm using the Operational Criteria for Psychosis (OPCRIT); this decreased the potential of subjective bias occurring [32].

#### 2.3.3. Social Dysfunction (SHIP Questions 10.01–10.10)

The SHIP measured social isolation as part of a social dysfunction assessment designed to capture via self-report: the nature of relationships with family and friends, the frequency of contact, the perceived need for friendships, availability of supportive relationships, maintenance of relationships, stigma and isolation.

Items used in the social dysfunction schedule are well accepted and were obtained from: the DIP-DM, SANE Australia Research Bulletins, the 2007 Survey of Mental Health and Wellbeing and the Stigma Shout Survey which are some of the most frequently used and well accepted measures [31,33,34,35,36]. The extent of social dysfunction was then rated by the trained interviewers based on responses given in areas mentioned above, leading to three classifications: those with ‘no dysfunction’, ‘obvious dysfunction’ or ‘severe dysfunction’ in the previous year.

Social dysfunction was converted into a dichotomous scale comprising the categories ‘no dysfunction’ and ‘obvious to severe dysfunction’, to compare differences between the two groups.

#### 2.3.4. Diet (SHIP Questions 16.01, 16.02, 16.04, 16.05 and 16.07)

Diet composition was assessed using the Short Diet Questions derived from the 1995 National Nutrition Survey (NNS) [37]. Diet assessment was retrospective, covering the 4-week period before the interview on items including; usual number of meal events per day, usual weekly breakfast consumption, average serves of fruits and vegetables per day and food security/whether or not participants had run out of food and not been able to buy more in the preceding 12 months prior to the interview. The short diet questions have not been comprehensively assessed for validity however when responses to the questions were compared to 24-h recall data, validity of questions differed in magnitude [38]. The validity of questions assessing number of meal events, usual fruit and vegetable intake were reported to be fair whereas the validity of the question assessing the frequency of breakfast consumption was considered poor [38]. The validity of the food security question was regarded as good however results need to be interpreted with caution because validity was assessed against socioeconomic status data and not other measures of food security [38].

### 2.4. Regression Model Outcomes

Regression model outcomes in this investigation included the adherence to the Australian Dietary Guidelines (ADGs) for fruit and vegetable intake which recommend an intake of five serves of vegetables and two serves of fruit a day for adults between 19 and 60 years hence outcomes were either ‘met recommendations’ or ‘did not meet recommendations’ [39]. Food security status was measured on a dichotomous scale with yes and no as possible outcomes. Meal events per day were measured on a continuous scale and responses ranged from 0 to 30 [40]. Finally, weekly breakfast consumption was measured on a continuous scale and responses ranged from 0 to 7 [40].

### 2.5. Measurement of Potential Confounding Variables

Potential confounding variables were chosen on the basis of having a statistically significant relationship with both the independent and dependent variables used in the logistic regressions.

All variables in this study were tested for this relationship in addition to other suitable variables from the SHIP. Variables that had a statistically significant relationship with independent and dependent variables include: income per fortnight, smoking, homelessness and accessibility to public transport.

#### 2.5.1. Income per Fortnight (SHIP Questions 11.08)

Income per fortnight was the net income after tax and included non-taxable allowances (see demographics section for more information).

#### 2.5.2. Smoking (SHIP Question 20.68.08)

Smoking status was measured using a question from the Fagerstrom Test for Nicotine Dependence [41]. Participants were asked if they had smoked in the previous 4 weeks and if their answer was yes, participants were asked to quantify the number of cigarettes smoked per day. Responses ranged from 0 to 60. Number of cigarettes smoked per day is a valid measure for nicotine dependence [41].

#### 2.5.3. Homelessness (SHIP Questions 3.02)

‘Homeless’ in the SHIP was defined as living on the streets, parks, deserted buildings or living in temporary shelters. Homelessness was measured by asking participants how many days in the previous 12 months they had been homeless. Participants were asked to quantify the number of days they were homeless and responses ranged from 0 to 365.

#### 2.5.4. Public Transport Accessibility (SHIP Question 3.22)

The accessibility to public transport was measured as a categorical variable. Participants were asked if they had access to public transport close to where they were living and possible responses were either yes or no.

### 2.6. Ethics

Ethics (Reference Number: HNE HREC 09/11/18/5.10) for the SHIP in New South Wales (NSW) was granted by the Human Research Ethics Committee in October 2009 and is valid until February 2018. The Declaration of Helsinki was adhered to during all steps of the study.

### 2.7. Statistical Analysis

Statistical analyses were conducted using the Statistical Package for the Social Sciences (SPSS) [42]. Descriptive statistics were performed for the following variables: demographics, social dysfunction, diet, diagnoses, smoking, homelessness and public transport accessibility. Frequencies were given for categorical variables and means, medians, standard deviations and interquartile ranges were given for continuous variables. Descriptive statistics then were split by social dysfunction for all variables to explore differences in characteristics between participants who did not have a social dysfunction and those who had an obvious to severe social dysfunction. To further evaluate the differences between the two social dysfunction groups, chi squared tests or one-way ANOVA tests were run depending on the suitability between the social dysfunction groups and the demographic variables to reveal any significant relationships.

Five binary logistic regression models were used to examine the independent association of social dysfunction with the dependent variables which were food security status, fruit intake, vegetable intake, number of meal events during the day and weekly breakfast consumption. Models were adjusted for homelessness, fortnightly income, public transport accessibility and smoking. Binary logistic regression assumptions were confirmed which include: a dependent variable measured on a dichotomous scale, presence of one or more independent variable and independence of observations [43].

A significance level of *p* < 0.05 was used for all analyses.

## 3. Results

### 3.1. Demographics

Participants from the HNE had a mean age of 38 (10.32) years, 60.6% were male and 5.4% were of Aboriginal or Torres Strait Islander descent. Most participants were single or never married (59.3%), had a diagnosis of schizophrenia (49.8%) and received a government payment as their main source of income (88.7%). Obvious to severe social dysfunction was observed in 62.4% of respondents. When frequencies were split by social dysfunction, 35.7% of people with obvious to severe social dysfunction were single or never married, 29.9% had a diagnosis of schizophrenia and 57.9% received a government pension as their main source of income. Comparatively, 23.5% of people with no dysfunction were single or never married, 19.9% had a diagnosis of schizophrenia and 30.8% received a government pension as their main income. Approximately one-fifth (22.6%) of study participants left school prior to obtaining a secondary school leaving certificate. Almost two thirds of participants had an income between $500 and $799 per fortnight (59.3%).

Most participants did not meet the recommendations for vegetable intake (86.9%) or for fruit intake (70.6%). One-quarter (25.3%) of all participants reported running out of food and not being able to buy more at least once in the previous 12 months. Participants consumed an average of 3.71 (1.43) meals/day, had breakfast on average 4.27 (3.06) times per week and smoked an average of 15.00 (14.82) cigarettes/day. Participants were also homeless on average 3.28 (26.54) days/year and 8.6% of participants did not have access to public transport. Upon splitting frequencies by social dysfunction, among people with obvious to severe social dysfunction, 17.2% left school prior to obtaining a secondary school leaving certificate, 38.0% had an income between $500 and $799, 52.9% did not meet vegetable intake requirements, 46.2% did not meet fruit intake recommendations and 14.0% ran out of food without being able to buy more. Similarly, among participants with obvious to severe social dysfunction, the average number of meals consumed per day was 3.96 (1.45), the average breakfast consumption was 3.97 (3.11) times per week and the average number of cigarettes smoked per day was 16.76 (15.64). Participants with obvious to severe social dysfunction had an average of 0.70 (5.42) homeless days and 5.9% did not have access to public transport. In comparison, 5.4% of people with no dysfunction left school prior to obtaining a secondary school leaving certificate, 21.3% had an income between $500 and $799, 33.9% did not meet vegetable intake requirements, 24.4% did not meet fruit intake recommendations and 11.3% ran out of food without being able to buy more. Additionally, participants with no social dysfunction consumed 3.81 (1.44) meals/day, had breakfast 4.76 (2.93) times per week and smoked 19.75 (16.63) cigarettes/day. People with no social dysfunction were homeless 2.31 (21.28) days/year and only 2.7% did not have access to public transport.

Participants with obvious to severe dysfunction were more likely to receive a government pension as the main source of income (*X* (1) = 7.112, *p* = 0.008) with 57.9% receiving payments compared to 30.8% of those with no dysfunction. Net income per fortnight was also significantly associated with social dysfunction (*X* (4) = 16.819, *p* = 0.002), however trends comparing those with no dysfunction to those with obvious to severe dysfunction are more difficult to establish because of the numerous income categories; the most apparent trends however revealed that 38.0% of participants with obvious to severe social dysfunction earned between $500–$799 per fortnight whereas 21.3% of participants with no dysfunction were in this wage bracket.

Analyses that were close to statistical significance included the test between social dysfunction and breakfast consumption (*F*(1,219) = 3.477, *p* = 0.064); participants with no dysfunction ate breakfast (on average) more occasions during the week (4.76 (2.93)) than those with obvious to severe social dysfunction (3.97 (3.11)). Similarly, the analysis between social dysfunction and number of cigarettes smoked (*F*(1,171) = 3.779, *p* = 0.054) showed that participants with no dysfunction were more likely to smoke more cigarettes (on average) 19.75 (16.64) than people with obvious to severe social dysfunction 15.00 (14.82) (Table 1).

### 3.2. Social Dysfunction and Diet

The unadjusted and adjusted analyses between social dysfunction and food security, fruit intake, vegetable intake and number of meal occasions were not statistically significant. The unadjusted analyses between social dysfunction and weekly breakfast consumption neared statistical significance (0.92 (95% CI (0.84–1.01)), *p* = 0.06). The adjusted model between social dysfunction and weekly breakfast consumption revealed that participants with obvious to severe social dysfunction were 0.87 less likely to eat breakfast than those with no social dysfunction ((95% CI (0.78–0.98)), *p* = 0.02) (Table 2).

## 4. Discussion

The study aimed to examine the association of social dysfunction with food security status, fruit intake, vegetable intake, breakfast consumption and meal frequency in people from the HNE catchment site of the SHIP. Social dysfunction was significantly associated with breakfast consumption in this study; those with no social dysfunction were 13% more likely to have breakfast than those with social dysfunction. This is a new finding as the relationship between breakfast consumption and social dysfunction has not been assessed previously; this outcome is however anticipated as people who are more socially isolated report practicing poorer dietary behaviours such as consuming fewer servings of fruit and vegetables [18,20]. Despite a statistically significant relationship between social dysfunction and breakfast consumption (0.87 (95% CI (0.78–0.98)), *p* = 0.02), other dietary outcomes such as fruit and vegetable intake, meal consumption and food security did not achieve a statistically important association with social dysfunction (*p* > 0.05). Frequencies however revealed that participants with obvious to severe social dysfunction had poorer compliance to fruit and vegetable intake recommendations and were more likely to suffer food insecurity therefore this relationship cannot be disregarded completely. Programs designed to foster social interaction particularly through communal meals may help promote positive dietary patterns [44].

Food insecurity in this cohort was not independently associated with social dysfunction even when the logistic regressions were adjusted for homelessness, smoking, public transport accessibility and fortnightly income. Nonetheless, structural factors related to social isolation such as low income, poor housing and transport inaccessibility may also impact food insecurity because of the financial interrelationship between the factors and the dependence of food security on financial stability [21,22,45]. The overwhelming majority of HNE participants received government payments as their main source of income, with incomes of almost two-thirds of the cohort falling between $500 and $799 per fortnight. Results revealed that participants with obvious to severe social dysfunction were more likely to receive government pensions as their main source of income and earn between $500–$799 per fortnight highlighting the interrelationship of social dysfunction and income. The mean average fortnightly income for the Australian general population at the time of the survey was $2522; Australians with psychosis were thus living on much less resources than the average Australian, which may increase their risk of food insecurity [21,46]. Despite not achieving statistical significance, frequencies revealed that participants with obvious to severe social dysfunction had a higher prevalence of food insecurity than those with no dysfunction and contributing factors could be low earnings evidenced by the increased likelihood of receiving government pensions as the primary income source. An increased risk of food insecurity may also predispose individuals to poorer fruit and vegetable intake, as foods with a higher calorie density are less expensive and have been shown to be the likely option in individuals suffering food insecurity [45].

Despite increased levels of food insecurity being reported in this cohort, this was still likely to be an underestimation because a single measure of food security was used that was only sensitive to food depletion [47]. Food insecurity actually spans from anxiety and uncertainty of food acquisition to an extremity where children forgo meals [47,48]. Therefore, the sensitivity of the tool can only partially explain the absence of associations between food insecurity and social dysfunction. Further, the food security question was retrospective for the previous 12 months and relied on self-report responses which depend on memory; a faculty which is reportedly impaired in people with schizophrenia [49]. Schizophrenia as a diagnosis had the highest prevalence in this population; it affected almost half of the participants which may have had a considerable effect on the accuracy of self-reported information [50]. More participants with obvious to severe social dysfunction had a diagnosis of schizophrenia and fared worse than those with no social dysfunction perhaps due to the illness [50]. To circumvent the shortcoming that was as a result of the limited efficacy of food security tool, future studies could use a more comprehensive tool or modify the existing tool to cover shorter time-periods and include other facets of food security. Several factors need to be considered in order to comprehensively evaluate food security [47]. These factors include: anxiety about food budget or food supply, running out of food due to financial incapacity, perceived inadequacy of food consumed, consuming less and cheaper foods than usual, reduced food intake and hunger or loss of weight in adults and eventually in children [47]. These factors need to be considered when designing food security tools in the future. Effective strategies that may be used to combat food insecurity involve targeting the environment, for example organised shopping trips which may also encourage increased fruit and vegetable consumption [51].

Poor intake of fruits and vegetables was the expected outcome due to similar reports in the overall SHIP study [30]. No significant association was found between social dysfunction and poor fruit and vegetable intake in the current study. More participants with obvious to severe social dysfunction than those with no dysfunction did not meet fruit recommendations (24.4% vs. 46.2%) and vegetable recommendations (33.9% vs. 52.9%). This trend was expected as results from a nationally representative cohort in the USA showed that participants with fewer social contacts had a lower healthy eating index score and were less likely to consume fruits and vegetables [18]. Conklin, Forouhi [19] and colleagues showed that infrequent social contact was associated with decreased consumption in quantity and variety of fruits and vegetables; it is possible that outcome discrepancies between the present study and previous research could be due to differential measures of social isolation and social contact being utilized between studies [22]. It is also of significance to note that previous investigations between diet and social relationships have focused on senior populations above the age of 50 years [1,19,52]. Similar studies where diet outcomes are analysed against social isolation are not available in people with psychosis. In view of this, more research needs to be focused on social isolation and its varied effects on all groups at risk.

Participants were less likely to meet vegetable intake recommendations than fruit intake recommendations. While the Short Diet Questions used here did not examine respondents’ preference for fruits versus vegetables, participants living with psychosis may have increased levels of apathy and disability which may hinder their ability to prepare a meal, perhaps explaining the higher compliance rates to the fruit intake recommendations [53,54,55]. Further, successfully estimating and reporting vegetable portion sizes with no visual reference may be complex for anyone and especially those with cognitive difficulty [56]. Fruit does not generally have this limitation.

## 5. Limitations

The findings of this study are subject to the cross-sectional design, hence cause and effect relationships cannot be assumed. Additionally, men represented about 60% of participants which may imply sampling bias; some studies seem to however suggest that incidence of various diagnoses characterised by psychosis may be higher in men however the evidence is inconclusive in long-term patients [57]. Given that social dysfunction and nutrition were one of many points of interest in the SHIP, data collected could have been more comprehensive if time and resources were ampler. Social dysfunction scores may have been subject to bias as they were based on interviewer judgment following a series of questions from standardised tools; this risk was however minimized by training interviewers and maintaining inter-rater reliability through testing at start, during and end of data collection. The measurement of social isolation is complex due to numerous subjective and objective factors that require consideration [22]. Social isolation has been measured using various indices, therefore comparing data may be difficult. Collapsing social dysfunction in the current study into a dichotomous scale allowed a clear comparison between those who did not have social dysfunction and those who had obvious to severe social dysfunction allowing us to establish various trends in this study, however a continuous scale of social dysfunction may have provided a richer analysis.

The Short Diet Questions utilised in the SHIP are a concise method of assessing dietary composition [58] and have been used widely in national surveys, therefore data are directly comparable to these studies. Nevertheless, the Short Diet Questions rely on retrospective, self-reported data; hence any bias would most likely be toward the null. To ameliorate this, trained interviewers were employed to administer the SHIP, and cue cards were utilised, showing pictorial illustrations of how much a serve of fruit or vegetable was consumed. Additionally, the Short Diet Questions have predetermined categories for the fruit and vegetable consumption, hence data collected were categorical, limiting evaluation. Food security prevalence may have also been underestimated due to poor sensitivity of the tool. Finally, the validity of the breakfast consumption question was regarded as poor for use in the general population, therefore results of this study need to be interpreted with caution [38].

## 6. Future Research Directions

Many of these limitations could be avoided in a prospective study where tools measuring diet outcomes are designed to suit people with psychosis and piloted to ensure validity and reliability of findings [59].

## 7. Conclusions

In summary, participants in this study living with psychosis have high rates of social dysfunction, significant levels of food insecurity, intakes of fruits and vegetables that are well below recommendations and breakfast consumption that is highly variable. Social dysfunction was not independently associated with food security status, fruit and vegetable intake and number of meal occasions in the current study. Social dysfunction was however found to have an independent negative association with frequency of breakfast consumption. Frequencies revealed that participants with obvious to severe social dysfunction fared worse not only in nutrition related outcomes, but also in the overall analysis undertaken in this study. This study highlighted the need to focus on dietary behaviour as well as social dysfunction in lifestyle interventions delivered to people with psychosis as both of these appear to be pressing issues in this population group. Further research is needed using more objective and elaborate tools to elucidate potential hidden relationships between social dysfunction and nutritional outcomes.

## Figures and Tables

**Table 1:** Demographics by Social Dysfunction.

		No Dysfunction (*n* = 83, 25.3% of *n* = 221) *n*% or M (SD) and Mdn (IQR)	Obvious to Severe Dysfunction (*n* = 138, 62.4% of *n* = 221) *n*% or M (SD) and Mdn (IQR)	HNE Participant Frequencies (*n* = 221) *n*% or M (SD) and Mdn (IQR)	Test
Sex	Male	53 (24.0%)	81 (36.7%)	134 (60.6%)	*X*^2^ = 0.578, *df* = 1, *p* = 0.447
	Female	30 (13.6%)	57 (25.8%)	87 (39.4%)	
Age		39.52 (10.23) 38.00 (15.00)	37.77 (11.14) 36.50 (16.00)	38.43 (10.82) 38.00 (16.00)	*F*(1,219) = 1.358, *p* = 0.245
Aboriginal/Torres Strait Islander descent	No	78 (35.3%)	131 (59.3%)	209 (94.6%)	*X*^2^ = 0.091, *df* = 1, *p* = 0.762
	Yes	5 (2.3%)	7 (3.2%)	12 (5.4%)	
Marital Status	Single/Never Married	52 (23.5%)	79 (35.7%)	131 (59.3%)	*X*^2^ = 1.875, *df* = 5, *p* = 0.866
	Married/De Facto	12 (5.4%)	26 (11.8%)	38 (17.2%)	
	Separated/Divorced	18 (8.1%)	29 (13.1%)	47 (21.3%)	
	Widowed	1 (0.5%)	4 (1.8%)	5 (2.3%)	
Diagnosis	Schizophrenia	44 (19.9%)	66 (29.9%)	110 (49.8%)	*X*^2^ = 3.974, *df* = 6, *p* = 0.680
	Schizoaffective	6 (2.7%)	20 (9.0%)	26 (11.8%)	
	Bipolar, mania	12 (5.4%)	19 (8.6%)	31 (14.0%)	
	Depressive psychosis	4 (1.8%)	3 (1.4%)	7 (3.2%)	
	Delusional disorders and other non-organic psychosis	6 (2.7%)	12 (5.4%)	18 (8.1%)	
	Severe depression without psychosis	9 (4.1%)	14 (6.3%)	23 (10.4%)	
	Screen-positive for psychosis but did not meet full criteria for ICD-10 psychosis	2 (0.9%)	4 (1.8%)	6 (2.7%)	
Government pension, allowance or benefit as the main source of income	No	13 (5.9%)	7 (3.2%)	20 (9.0%)	*X*^2^ = 7.112, *df* = 1, *p* = 0.008 ***
	Yes	68 (30.8%)	128 (57.9%)	196 (88.7%)	
Highest qualification obtained	Left school no qualifications	12 (5.4%)	38 (17.2%)	50 (22.6%)	*X*^2^ = 14.15, *df* = 5, *p* = 0.225
	Secondary school qualification/leaving certificate	15 (6.8%)	28 (12.7%)	43 (19.5%)	
	Tertiary Certificates	44 (19.9%)	62 (28.1%)	106 (48.0%)	
	Bachelor’s Degree	5 (2.3%)	4 (1.8%)	9 (4.1%)	
	Postgraduate Qualifications	2 (0.9)	1 (0.5%)	3 (1.4%)	
	Other specify	5 (2.3%)	5 (2.3%)	10 (4.5%)	
Income per Fortnight	Less than $300 per fortnight	3 (1.4%)	2 (0.9%)	5 (2.3%)	*X*^2^ = 16.819, *df* = 4, *p* = 0.002 ***
	Between $300–$499 per fortnight	4 (1.8%)	10 (4.5%)	14 (6.3%)	
	Between $500–$799 per fortnight	47 (21.3%)	84 (38.0%)	131 (59.3%)	
	Between $800–$1000 per fortnight	11 (5.0%)	28 (12.7%)	39 (17.6%)	
	More than $1000 per fortnight	15 (6.8%)	4 (1.8%)	19 (8.6%)	
Vegetables consumed (no of serves per day in the last 4 weeks)	Did not meet recommendations (≤0–3 serves/day)	75 (33.9%)	117 (52.9%)	192 (86.9%)	*X*^2^ = 1.415, *df* = 1, *p* = 0.234
	Met recommendations (≥4–5 serves/day)	8 (3.6%)	21 (9.5%)	29 (13.1%)	
Fruit consumed (no of serves per day in the last 4 weeks)	Did not meet recommendations (≤1 serve per day)	54 (24.4%)	102 (46.2%)	156 (70.6%)	*X*^2^ = 1.956, *df* = 1, *p* = 0.162
	Met recommendations (≥2–3 serves per day)	29 (13.1%)	36 (16.3%)	65 (29.4%)	
Ran out of food (last 12 months)	No	58 (26.2%)	107 (48.4%)	165 (74.7%)	*X*^2^ = 1.606, *df* = 1, *p* = 0.205
	Yes	25 (11.3%)	31 (14.0%)	56 (25.3%)	
Meal events (average number per day in the last 4 weeks)		3.81 (1.44) 4.00 (2.00)	3.96 (1.45) 4.00 (2.00)	3.71 (1.43) 4.00 (2.00)	*F*(1,219) = 1.612, *p* = 0.206
Breakfast consumption (average number of times per week in the last 4 weeks)		4.76 (2.93) 7.00 (5.00)	3.97 (3.11) 5.00 (7.00)	4.27 (3.06) 7.00 (7.00)	*F*(1,219) = 3.477, *p* = 0.064
Number of cigarettes smoked (per day in the last 4 weeks)		19.75 (16.63) 19.00 (23.75)	16.76 (15.64) 12.00 (25.00)	15.00 (14.82) 15.00 (24.00)	*F*(1,171) = 3.779, *p* = 0.054
No of homeless days (in the last 12 months)		2.31 (21.28) 0.00 (0.00)	0.70 (5.42) 0.00 (0.00)	3.28 (26.54) 0.00 (0.00)	*F*(1,218) = 0.131, *p* = 0.718
Accessibility to public transport in location of residence	No	6 (2.7%)	13 (5.9%)	19 (8.6%)	*X*^2^ = 0.283, *df* = 1, *p* = 0.595
	Yes	74 (33.5%)	122 (55.2%)	196 (88.7%)	

Data given as *n* (%) unless otherwise stated; ** *p* < 0.05; *** *p* < 0.01. ICD: International Statistical Classification of Diseases and Related Health Problems, M (SD): mean (standard deviation), Mdn (IQR): median (interquartile range), *X^2^*: chi squared tests, *df*: degrees of freedom.

**Table 2:** Binary Logistic Regressions for Social Dysfunction and Food Security, Fruit Intake, Vegetable Intake, Meal Occasions and Weekly Breakfast Consumption.

Independent Variable	Dependent Variable	Unadjusted Odds Ratio, 95CI and (*p*-Value)	Adjusted Odds Ratio *, 95% CI and (*p*-Value)
Social Dysfunction	Food Security	1.49	1.29
		0.80–2.76	0.62–2.67
		(0.21)	(0.50)
	Fruit Intake	1.52	0.81
		0.84–2.75	0.39, 1.67
		(0.16)	(0.56)
	Vegetable Intake	0.59	1.20
		0.25–1.41	0.47, 3.05
		(0.24)	(0.70)
	Meal Occasions	0.88	0.92
		0.73–1.07	0.73, 1.17
		(0.21)	(0.51)
	Weekly Breakfast Consumption	0.92	0.87
		0.84–1.01	0.78, 0.98
		(0.06)	(0.02) **

* Adjusted for homelessness, smoking, public transport, current net fortnightly income; ** *p* < 0.05.
